# A convenient clinical nomogram for predicting the cancer-specific survival of individual patients with small-intestine adenocarcinoma

**DOI:** 10.1186/s12885-020-06971-6

**Published:** 2020-06-01

**Authors:** Na Wang, Jin Yang, Jun Lyu, Qingqing Liu, Hairong He, Jie Liu, Li Li, Xuequn Ren, Zhendong Li

**Affiliations:** 1 Department of Clinical Research, The First Affiliated Hospital of Jinan University, Guangzhou, Guangdong Province China; 2 School of Nursing and Health, Henan University, Kaifeng, Henan China; 3 School of Public Health, Xi’an Jiaotong University Health Science Center, Xi’an, Shaanxi China; 4 Clinical Research Center, The First Affiliated Hospital of Xi’an Jiaotong University, Xi’an, Shaanxi China; 5 Center for Evidence-Based Medicine and Clinical Research, Huaihe Hospital of Henan University, Kaifeng, Henan China; 6 Department of General Surgery, Huaihe Hospital of Henan University, Kaifeng, Henan China; 7 Department of Hepatobiliary Surgery, The First Affiliated Hospital of Jinan University, Guangzhou, Guangdong Province China

**Keywords:** Small-intestine adenocarcinoma, Nomogram, Cancer-specific survival

## Abstract

**Background:**

The objective of this study was to develop a practical nomogram for predicting the cancer-specific survival (CSS) of patients with small-intestine adenocarcinoma.

**Methods:**

Patients diagnosed with small-intestine adenocarcinoma between 2010 and 2015 were selected for inclusion in this study from the Surveillance, Epidemiology, and End Results (SEER) database. The selected patients were randomly divided into the training and validation cohorts at a ratio of 7:3. The predictors of CSS were identified by applying both forward and backward stepwise selection methods in a Cox regression model. The performance of the nomogram was measured by the concordance index (C-index), the area under receiver operating characteristic curve (AUC), calibration plots, the net reclassification improvement (NRI), the integrated discrimination improvement (IDI), and decision-curve analysis (DCA).

**Results:**

Multivariate Cox regression indicated that factors including age at diagnosis, sex, marital status, insurance status, histology grade, SEER stage, surgery status, T stage, and N stage were independent covariates associated with CSS. These factors were used to construct a predictive model, which was built and virtualized by a nomogram. The C-index of the constructed nomogram was 0.850. The AUC values indicated that the established nomogram displayed better discrimination performance than did the seventh edition of the American Joint Committee on Cancer TNM staging system in predicting CSS. The IDI and NRI also showed that the nomogram exhibited superior performance in both the training and validation cohorts. Furthermore, the calibrated nomogram predicted survival rates that closely corresponded to actual survival rates, while the DCA demonstrated the considerable clinical usefulness of the nomogram.

**Conclusion:**

We have constructed a nomogram for predicting the CSS of small-intestine adenocarcinoma patients. This prognostic model may improve the ability of clinicians to predict survival in individual patients and provide them with treatment recommendations.

## Background

Adenocarcinoma, neuroendocrine tumors, stromal tumors, and lymphoma are the four main histological types of small-intestine malignancies, of which small-intestine adenocarcinoma is the most-common type, accounting for 30 to 40% of these tumors [1, 2]. The rarity of small-intestine adenocarcinoma restricts studies of its treatment and prognosis. Moreover, the rationale for the choice of therapeutic regimen of small-intestine adenocarcinoma are extrapolated from colon adenocarcinoma, with which it shares many clinicopathologic features and similar molecular alterations that lead to carcinogenesis [3]. There has been far less research into the prognosis of small-intestine adenocarcinoma compared to colorectal cancer.

The most commonly used small-intestine adenocarcinoma prediction system is the American Joint Committee on Cancer (AJCC) TNM staging system, which is based on the tumor infiltration depth (T), number of metastatic lymph nodes (N), and distant metastasis (M). Although the TNM system is widely used to estimate prognoses and determine clinical treatments of cancer patients, this system might not adequately encompass the tumor biology and therefore be unreliable for predicting outcomes of small-intestine adenocarcinoma [4]. Furthermore, other clinicopathological features such as age of diagnosis, marriage status, tumor location, and surgery status also affect the prognosis [5–7].

Nomograms have been widely used as a valuable pictorial tool for combining biological and clinical variables in the construction of prognostic models using traditional statistical methods such as a Cox proportional-hazards regression model to determine the prognosis of multiple malignancies [8–10]. In addition, several studies have shown that a nomogram is superior to the AJCC TNM staging system in predicting the survival of cancer patients [11–14]. However, few studies have used nomograms to predict the prognosis of patients with small-intestine adenocarcinoma. This study therefore aimed to construct a clinical nomogram for predicting the 3- and 5-year cancer-specific survival (CSS) in small-intestine adenocarcinoma patients based on a cohort from the Surveillance, Epidemiology, and End Results (SEER) database.

## Methods

### Study cohorts

The SEER database covers approximately 28% of the US population and contains a large amount of evidence-based medical data [15]. We selected cases of small-intestine adenocarcinoma using the newest database of SEER* Stat (version 8.3.5; https://seer.cancer.gov/): ‘Incidence–SEER 18 Regs Research Data + Hurricane Katrina Impacted Louisiana Cases, Nov 2017 Sub (1973–2015 varying)’. The patients were identified using the following SEER variables: “Site Recode ICD-O-3 / WHO 2008 classification” (small intestine) and “Histology recode – broad groupings” (histology codes: 8140–8389). The TNM staging data were extracted according to the code ‘derived AJCC TNM stage group 7th ed**’** (henceforth simply referred to as the AJCC staging system); this was published in 2010, and so the allowable year of diagnosis ranged from 2010 to 2015. Survival information was extracted using the codes ‘SEER cause-specific death classification’ and ‘survival months’. Patients were excluded if their first primary tumor was not small-intestine adenocarcinoma, were younger than 18 years, or their information was unknown or ambiguous.

Our screening of the SEER database identified 4971 eligible patients. Random numbers generated using R software were used to randomly allocate 70% (*n* = 3479) of these patients to a training cohort for developing the nomogram, with the remaining 30% (*n* = 1492) of the patients used as a validation cohort. This study was conducted in accordance with the SEER data-use agreement, and was approved by the institutional research committee of the First Affiliated Hospital of Jinan University.

### Study variables and endpoint

The characteristics of the patients analyzed were age at diagnosis, sex, race, marital status, insurance status, histology grade, AJCC stage, treatment status (for surgery, chemotherapy, and radiotherapy), and survival time. The marital status was dichotomized into married (including domestic partnership and common-law marriage) and unmarried (including single, separated, divorced, or widowed). The primary endpoint was the CSS time, which was defined as the period from the date of diagnosis to the date of death attributed to small-intestine adenocarcinoma.

### Statistical analyses

The method was performed as our previous study described [16]. Briefly, Fisher’s exact or Chi-square tests for categorical variables and Student’s t-test for continuous variables were calculated to compare baseline characteristics. A nomogram was developed based on the independent prognostic factors determined by applying both forward and backward stepwise selection methods in a Cox regression model. The performance of the nomogram was measured by concordance index (C-index) and assessed by calibration curves. The precision of the 3- and 5-year survival of the nomograms was evaluated and compared using the area under receiver operating characteristic (ROC) curve (AUC). Furthermore, we determined the improvement that the new prediction model represented over the AJCC staging system in both the training and validation cohorts using the net reclassification improvement (NRI) and the integrated discrimination improvement (IDI). Finally, we used decision-curve analysis (DCA) to test the clinical applicability of the predictive model. A two-sided probability value of *P* ≤ 0.05 was considered to be statistically significant. All analyses were performed using R software (version 3.5.1; http://www.r-project.org).

## Results

### Patient characteristics

The baseline characteristics of the included patients are presented in Table 1. The median age at diagnosis was 63 years (25th–75th percentiles 54–72 years), and most of the patients were male, white, married, and insured. The most-common tumor grade was well-differentiated (*n* = 2962, 59.6%), while stage III (*n* = 2108, 42.4%) was the most common AJCC stage. There were 4525 (91.0%), 111 (2.2%), and 897 (18.0%) patients who received surgery, radiotherapy, and chemotherapy, respectively.

**Table 1:** Patient characteristics in the study

Variable	Total (*n* = 4971)	Training Cohort (*n* = 3479)	Validation Cohort (*n* = 1492)	*P*
Age, median (25th–75th percentile	63(54–72)	63(54–72)	63(54–72)	0.821
Sex n (%)				0.475
Male	2597 (52.2)	1806 (51.9)	791 (53.0)	
Female	2374 (47.7)	1673 (48.1)	701 (47.0)	
Race n (%)				0.832
White	3929 (79.0)	2751 (79.2)	1173 (78.6)	
Black	824 (16.6)	574 (16.5)	250 (16.7)	
Other	218 (4.4)	149 (4.3)	69 (4.6)	
Marital status n (%)				0.895
Married	3135 (63.1)	2192 (63.0)	943 (63.2)	
Unmarried	1836 (36.9)	1287 (37.0)	549 (36.8)	
Grade n (%)				0.143
I	2962 (59.6)	2080 (59.8)	882 (59.1)	
II	1412 (28.4)	1000 (28.7)	412 (27.6)	
III	565 (11.4)	374 (10.7)	191 (12.8)	
IV	32 (0.6)	25 (0.7)	7 (0.5)	
AJCC TNM stage n (%)				0.062
I	729 (14.7)	541 (15.5)	188 (12.6)	
II	1024 (20.6)	707 (20.0)	317 (21.2)	
III	2108 (42.4)	1459 (41.9)	649 (43.5)	
IV	1110 (22.3)	772 (22.2)	338 (22.6)	
Insurance Status				0.279
Insured	4320 (86.9)	3038 (87.3)	1282 (85.9)	
Medicaid	497 (10.0)	341 (9.8)	156 (10.4)	
Uninsured	154 (3.1)	100 (2.9)	54 (3.6)	
Surgery				0.199
Yes	4525 (91.0)	3155 (90.7)	1370 (91.8)	
No	446 (9.0)	324 (9.3)	122 (8.2)	
Radiation				0.628
Yes	111 (2.2)	80 (2.3)	31 (2.1)	
No	4860 (97.7)	3399 (97.7)	1461 (97.9)	
Chemotherapy				0.432
Yes	897 (18.0)	618 (17.8)	279 (18.7)	
No	4074 (81.9)	2861 (82.2)	1213 (81.2)	

### Nomogram construction

**Fig. 1:**
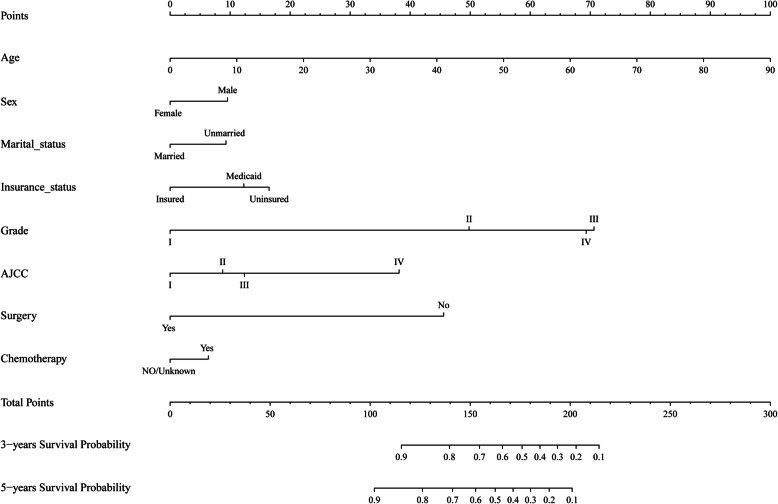
Nomogram predicting the 3- and 5-year cancer-specific survival (CSS) of patients with small-intestine adenocarcinoma

The following eight independent prognostic variables were found to be associated with CSS after performing both forward and backward stepwise selection in the Cox regression model in the training cohort: age at diagnosis, sex, marital status, insurance status, histology grade, AJCC TNM stage, surgery status, chemotherapy (Table 2). The nomogram established for predicting the 3- and 5-year CSS included variables that were significantly associated with CSS in the training cohort. The nomogram in Fig. 1 showed that age at diagnosis is the greatest contributor to the prognosis, followed by histology grade, surgery status, AJCC TNM stage, insurance status, sex, marital status and chemotherapy. The survival probability of an individual patient can be easily calculated by adding their scores for all of the selected variables.

**Table 2:** Selected variables by multivariate Cox regression analysis

characteristic	Multivariate analysis		
	HR	95%CI	*P*
Age	1.035	1.028–1.043	< 0.001
Sex			
Male	Reference		
Female	0.742	0.625–0.880	< 0.001
Marital status			
Married	Reference		
Unmarried	1.337	1.121–1.595	0.001
Grade			
I	Reference		
II	4.731	3.774–5.931	< 0.001
III	9.060	7.015–11.700	< 0.001
IV	8.670	4.383–17.267	< 0.001
AJCC TNM stage			
I	Reference		
II	1.314	0.928–1.862	0.124
III	1.472	1.050–2.062	0.025
IV	3.289	2.385–4.535	< 0.001
Insurance			
Insured	Reference		
Medicaid	1.467	1.128–1.909	0.004
Uninsured	1.673	1.072–2.612	0.024
Surgery			
Yes	Reference		
No	4.147	3.377–5.092	< 0.001
Chemotherapy			
Yes	Reference		
No	0.820	0.677–0.992	0.04

*HR* Hazard ratio; *CI* Confidence interval

### Validation and calibration of the nomogram

The C-index values of the nomogram and AJCC staging system for CSS in the training cohort were 0.850 and 0.643, respectively. Compared with the AJCC staging system, the nomogram showed enhanced discrimination for CSS prediction in the training cohort. Significant differences in C-index values for CSS were also found in the validation cohort, being 0.850 for the nomogram and 0.613 for the AJCC staging system.

**Fig. 2:**
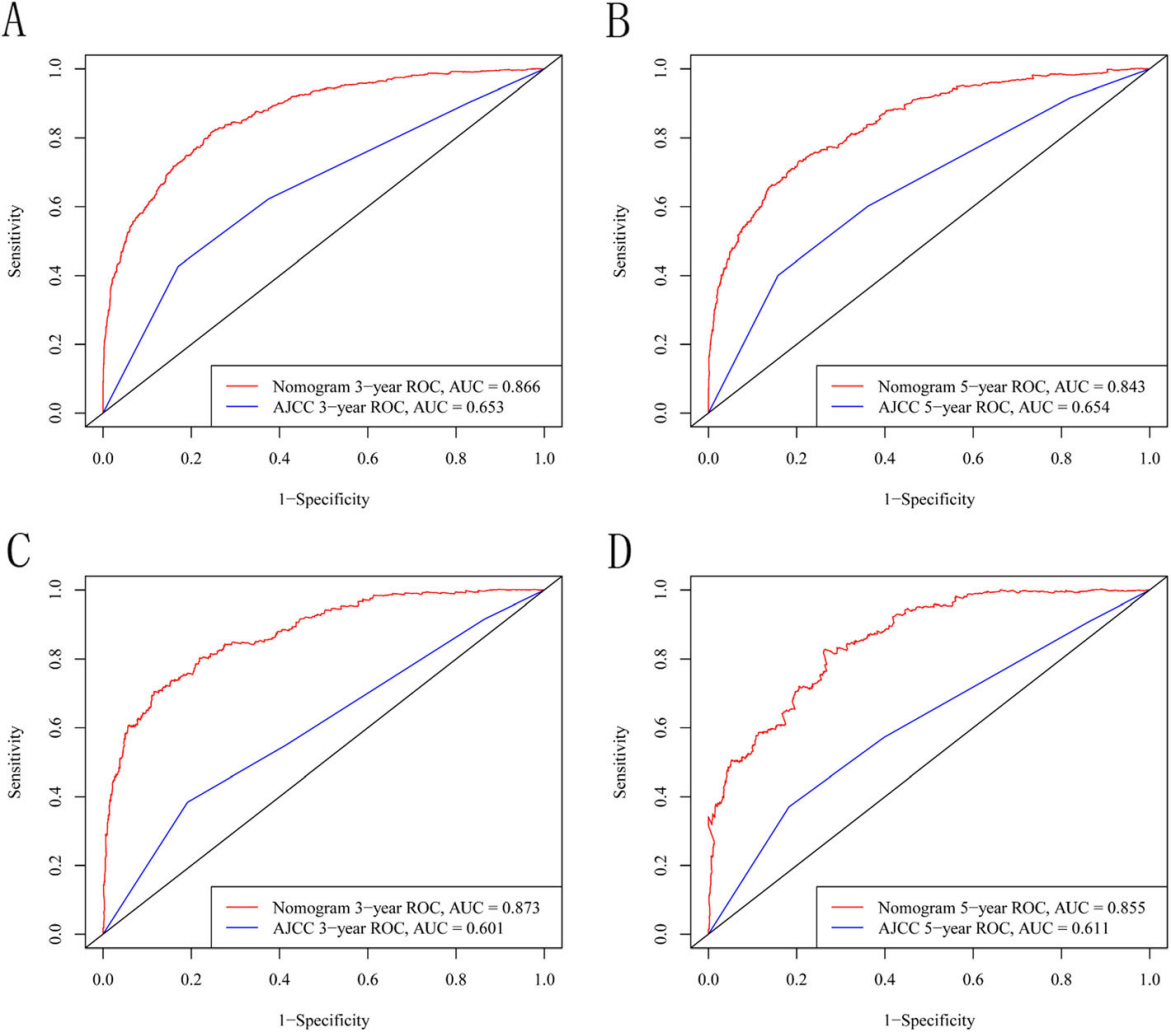
Coparison of the AUCs of the nomogram and 7th AJCC TNM staging system to prediction of CSS in the training set (**a**: 3 years; **b**: 5 years) and the validation set (**c**: 3 years; **d**: 5 years)

**Fig. 3:**
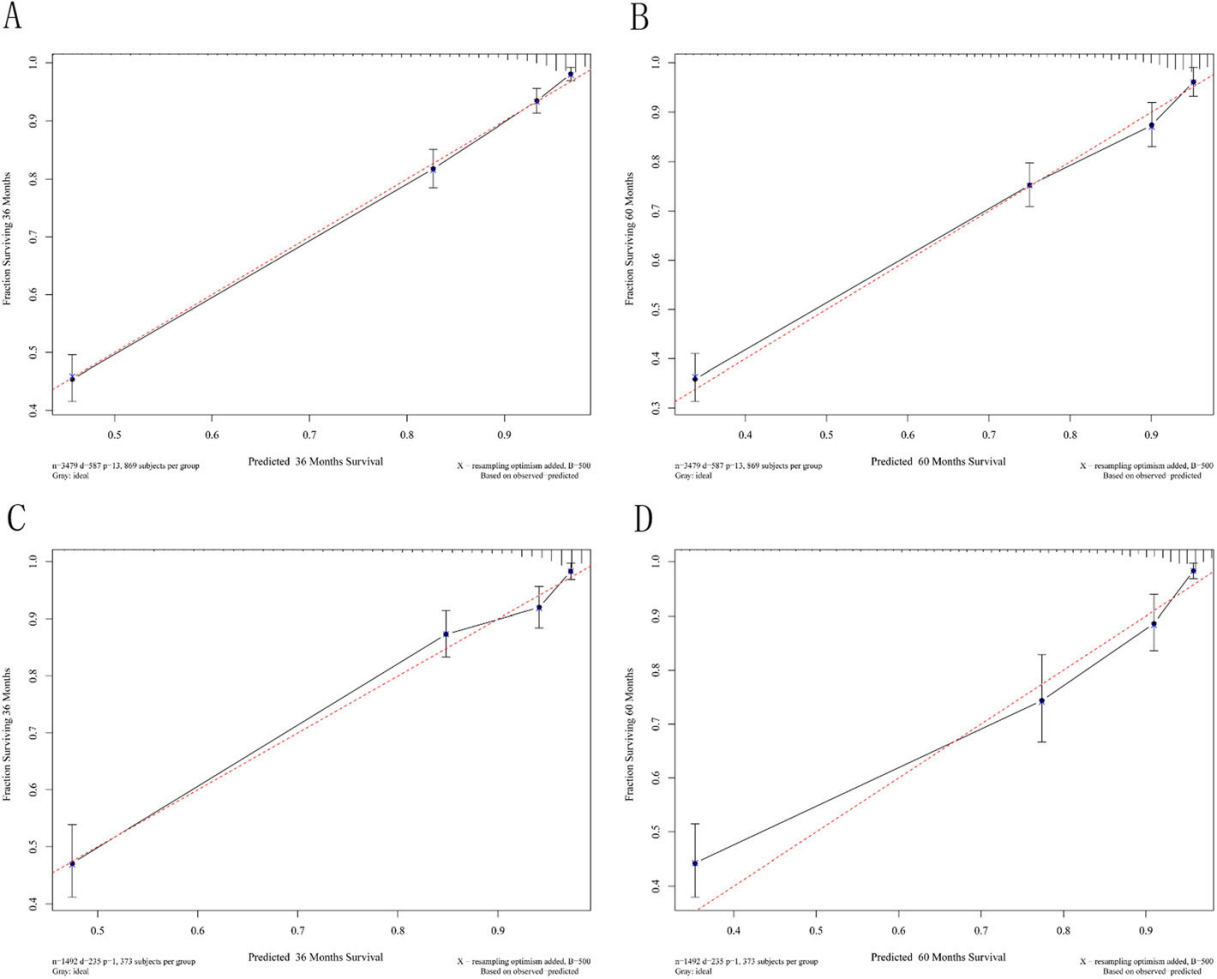
The calibration of the nomograms using the training set and validation set. (**a**) 3-year cancer-specific survival (CSS) and (**b**) 5-year CSS according to the training set. (**c**) 3-year CSS and (**d**) 5-year CSS according to the training set

The two AUC models of the 3- and 5-year CSS rates regarding the prediction ability of the two data sets were compared (Fig. 2). For the training cohort, the AUCs for predicting the 3- and 5-year CSS rates were 0.866 and 0.843, respectively, for the nomogram, and 0.653 and 0.654 for the AJCC staging system; the corresponding values in the validation cohort were 0.873, 0.855, 0.601, and 0.611, respectively. Figure 2 shows that the nomogram exhibited superior survival predictive ability compared to the AJCC staging system. Calibration plots of the nomogram show that the predicted 3- and 5-year survival probabilities for the training and validation cohorts were almost identical to the actual observations, especially for the 3-year CSS (Fig. 3).

The NRI values for the 3- and 5-year CSS rates were 100.4 (95% CI = 89.9–110.1%) and 91.7% (95% CI = 78.9–101.6%), respectively, in the training cohort, and 105.5% (95% CI = 90.9–120.6%) and 90.1% (95% CI = 76.1–109.0%) in the validation cohort. These results indicate the significant superiority of the predictive performance of the nomogram. Similarly, the IDI values for the 3- and 5-year CSS rates were 27.6 and 30.0%, respectively, in the training cohort, and 30.6 and 32.1% in the validation cohort (all *P* < 0.001). These results further demonstrate the improved predictive performance of the nomogram.

**Fig. 4:**
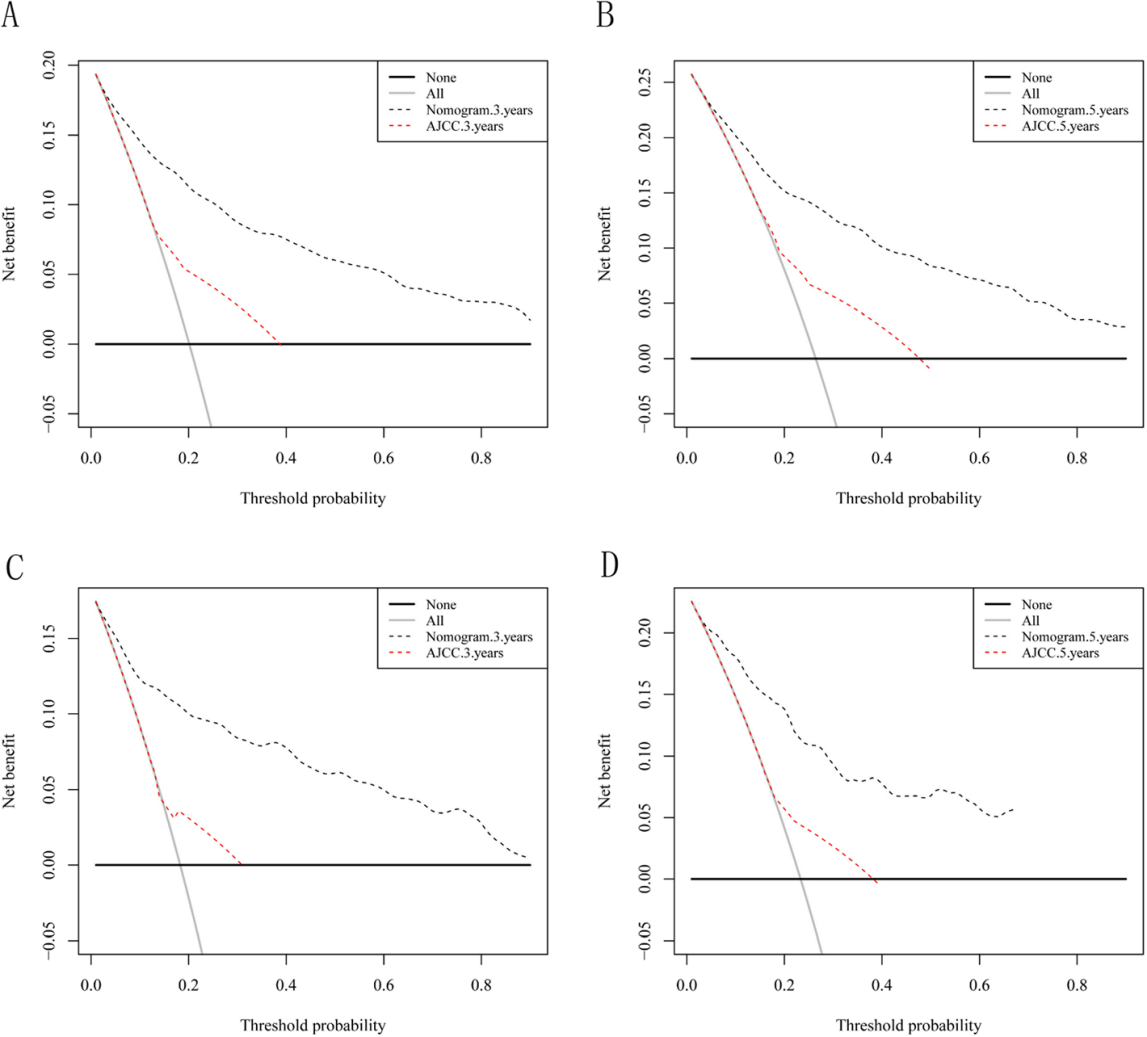
Decision curve analysis of the training set (**a** and **b**) and the validation set (**c** and **d**). The x-axis is the threshold probability, the y-axis is the net benefit rate. The black horizontal line indicates that cancer-specific death occurred in no patients. The gray oblique line indicates that all patients will have cancer specific death. The dashed line indicates the net benefit

Finally, DCA was used to compare the clinical validity of the nomogram to that of the AJCC staging system. A plot of the 3- and 5-year DCA curves with the threshold probability as the abscissa and the net benefit as the ordinate graphically indicate that compared with the AJCC staging system, the nomogram showed a larger net benefit across the range of death risks both in the training and validation cohorts (Fig. 4).

## Discussion

Small-intestine adenocarcinoma is a rare tumor that has a very low incidence compared with colorectal cancer. The smallness of the available samples associated with this rarity restricts data analyses and the ability to draw generalized conclusions in studies of small-intestine adenocarcinoma, which means that the relevant prognosis factors are still controversial [17, 18]. Nomograms have been widely used in recent years for predicting individualized survival outcomes in cancer patients [19–23]. The rarity of small-intestine adenocarcinoma increases the practicality of using a brief nomogram to predict patient survival in clinical decision-making.

Prognostic factors closely associated with CSS in small-intestine adenocarcinoma were included in the construction of the present nomogram, which was used to predict the 1- and 3-year CSS for small-intestine adenocarcinoma. Compared with the AJCC staging system, the nomogram shows better predictive performance, with a C-index of 0.858. Furthermore, the ROC curve, NRI, and IDI also demonstrated that the nomogram showed better predictive ability than the AJCC staging system. Moreover, the calibration curves indicated that the predicted 3- and 5-year CSS rates for the training and validation cohorts were almost identical to the actual observations, especially for the 3-year CSS. Finally, the DCA curves showed that the nomogram exhibits better clinical usefulness for predicting survival compared to the AJCC staging system.

The proposed nomogram contains several independent prognostic factors—age at diagnosis, sex, marital status, insurance status, histology grade, AJCC TNM stage, surgery status, chemotherapy—that were selected by applying both forward and backward stepwise selection methods in a Cox regression model. We found that increasing age was associated with worse survival outcomes, which is consistent with previous findings [3]. Some studies have suggested that small-intestine adenocarcinoma is more prevalent in males than in females [24]. We similarly found that the proportion of male patients in our study cohort was higher than that of females (52.2% vs 47.7%), and that being female appeared to be a protective factor for the CSS of small-intestine adenocarcinoma. In addition, we included insurance status in our analysis, and found that Medicaid and uninsured patients had an increased risk of death compared to insured patients, this result is consistent with our previous findings [25].

Surgery remains the most commonly used treatment method for small-intestine adenocarcinoma [1, 26], and it has been shown that radiation can also play a role in improving survival outcomes in these patients [1, 27, 28]. However, our research did not find this association, the main reason being that the current data on radiotherapy in the SEER database have potential bias because many factors that influence the course of treatment are not captured in the registry data. In addition, our research showed that chemotherapy was associated with poor survival, which may be because chemotherapy is main treatment strategies for unresectable stage IV small bowel adenocarcinoma [29], and the worse survival is caused by advanced disease.

Our nomogram highlights the significant contribution of histology grade, which is consistent with previous studies showing that histology grade is an independent predictor of survival [7, 30, 31]. In addition, there was no definitive correlation between tumor differentiation and tumor size or lymph node metastasis, while the last one is an important component of the TNM staging system. Even two patients at the same TNM stage can exhibit different scores for CSS on our nomogram based on their degrees of tumor differentiation. This may also be the reason why the survival predictions of our nomogram are superior to using the AJCC staging system.

Besides the factors mentioned above, distant metastasis and increased tumor and lymph node metastasis are risk factors for survival in small-intestine adenocarcinoma, as found previously [7, 31–33]. Moreover, this study also showed that not being married is associated with a poor prognosis of small-intestine adenocarcinoma, which is consistent with the findings of our previous study [34].

This study was subject to several limitations. First, although 10 variables were involved, only 8 of them are included in the nomogram, and there are also many other variables such as comorbidities, chemotherapy drugs, and molecular factors not included in the SEER database that might affect patient survival. Second, although we randomly divided the patients into the training and validation cohorts (at a ratio of 7:3) to evaluate the nomogram both internally and externally, it remains necessary to assess the accuracy of the model based on external validation in other populations. Third, the SEER database is retrospective and we excluded patients with incomplete information, which may have led to selection bias. Finally, although the DCA demonstrated the superiority of our nomogram over the AJCC staging system with a greater net benefit, this analysis is not absolutely accurate and so should only be used as reference information in clinical decision-making.

## Conclusion

In conclusion, we have developed and validated a nomogram for predicting the 3- and 5-years CSS rates of small-intestine adenocarcinoma patients. The nomogram is brief and convenient, and it exhibited a superior survival predictive ability compared to the AJCC staging system. The nomogram might assist clinicians in making predictions about the survival of individual patients and provide improved treatment recommendations.

## Data Availability

The data were abstracted from the Surveillance, Epidemiology, and End Results (SEER) database. This is an open database. (https://seer.cancer.gov).

## Abbreviations

CSS: Cancer-specific survival
SEER: Surveillance, epidemiology, and end results
C-index: Concordance index
ROC: Receiver operating characteristic
AUC: Area under receiver operating characteristic curve
NRI: Net Reclassification improvement
IDI: Integrated discrimination improvement
DCA: Decision-curve analysis
AJCC: American joint committee on cancer
SD: Standard deviation
HR: Hazard ratio
CI: Confidence interval
